# Role of microRNAs in chronic hepatitis E viral infection

**DOI:** 10.6026/973206300210240

**Published:** 2025-02-28

**Authors:** Zoya Shafat, Murshad Ahmed, Anam Farooqui, Nazim Khan, Shama Parveen

**Affiliations:** 1 Department of Interdisciplinary Research in Basic Sciences, Jamia Millia Islamia, New Delhi, India

**Keywords:** Hepatitis E virus, chronic hepatitis E virus infection, network topology, module detection, mRNA-miRNA network, key miRNAs, enrichment analysis

## Abstract

Hepatitis E virus (HEV) is an emerging zoonotic pathogen and it is associated with chronic hepatitis E virus infection (CHE) in immuno-suppressed patients due to failure of viral clearance. A network of the CHE-associated-differentially expressed genes (DEGs) is known. Hence, a comprehensive assessment of the known protein-protein interaction (PPI) network is of interest. After network clustering, the hub gene-associated microRNAs (miRNAs) were explored and subsequently, these identified miRNAs (miR-129-2-3p, miR-130a-3p, miR-138-5p, miR-212-3p, miR-221-3p, miR-27b-3p and miR-29c-3p) were undertaken for enrichment analysis. It should be noted that these miRNAs are significantly associated with Hepatitis E virus infection for further validation and analysis.

## Background:

Hepatitis E virus (HEV) is an RNA virus constituting a single (positive)-stranded linear genome [1]. Hepatitis E virus, causing Hepatitis E (liver disease), has become a major health concern globally. It is an important cause of waterborne acute hepatitis in adults in developing countries [2, 3]. In immunosuppressed patients, *i.e.*, hepatitis E virus patients receiving solid-organ transplant, failure of viral clearance has been reported leading to chronic hepatitis E virus infection (CHE) [4]. Chronic hepatitis E virus infection infections are persistent viral infections as hepatitis E virus RNA lasts for more than six months in the patients. Further, it was recognized that half of the recipients receiving kidney transplantation infected with hepatitis E virus develop chronic hepatitis E virus infection [5]. Chronic hepatitis E virus infection has been well documented in SOT recipients; therefore, hepatitis E virus induced chronic hepatitis should be studied intensively. Hepatitis E virus infection evolution toward chronic hepatitis E virus infection appears to be determined by the patient's immunological status. In SOT recipients, the development of chronic hepatitis E virus infection has been linked to the type or dose of immunosuppressive drugs received [5, 6], however, one-third chronic hepatitis E virus infection patients show hepatitis E virus clearance on the reduction in immunosuppressive drug doses [5]. In organ transplant recipients, chronic hepatitis E virus infection is accompanied with impaired hepatitis E virus specific T-cell responses [7]. Owing to inadequate information on hepatitis E virus prevalence and its impact in kidney transplant patients, a more detailed study is the need of the hour. The miRNAs (small non-coding RNAs) serve as direct antiviral entity for their host cells and the host's miRNAs are required by viruses in order to facilitate their survival and replication [8]. The potential miRNAs can be therapeutically used through targeting various steps in the hepatic pathophysiology. The miRNA expression is specifically altered in majority of liver diseases (both acute and chronic) [9]. Investigations have revealed the role of various signature miRNAs in hepatitis viruses, such as, hepatitis B and hepatitis C, by acting as potential biomarkers in its diagnosis and prognosis [10, 11] besides diverse diseases, such as sepsis [12] and NSCLC [13]. In this context, we identified the hub genes associated miRNAs by constructing the mRNA-miRNA construction network. Our previous study [14] has identified the network of the chronic hepatitis E virus infection -associated differentially expressed genes (DEGs), hereby, in this study; we further used the obtained network for downstream analysis. The sub network or community or modules were extracted from the primary network which was further analyzed. Our identified hub gene associated miRNAs could be implicated in chronic hepatitis E virus infection treatment.

## Materials and Methods:

## Methods flowchart:

The workflow of the integrative network-based method used for the present analysis is illustrated in Figure 1 (see PDF).

## Microarray data retrieval:

The gene expression dataset GSE36539 was retrieved from the GEO database (https://www.ncbi.nlm.nih.gov/geo/) of NCBI [15, 16].

## Dataset selection criteria:

The database housed in NCBI was searched for hepatitis E virus associated microarray data using appropriate keywords: "mRNA expression," "Hepatitis E Virus or hepatitis E virus" and "Homo sapiens" (organism). The following inclusion criteria were applied for the appropriate selection of dataset. (i) Consist of control and infected samples, (ii) Specifically containing samples representing all stages of infection (mild, moderate and severe) and (iii) Analysis of mRNA expression profiling. This approach ensured that our analysis encompassed the full spectrum of infection severity, facilitating a comprehensive examination of gene expression changes associated with different stages of hepatitis E virus infection.

## Patients:

Our study included a total of 16 kidney transplant recipients/patients of whom 8 were suffering from chronic hepatitis E virus infection (CHE patients) and 8 were control patients (healthy individuals without viral infection). We used GSE36539 for the present analysis submitted by Moal and colleagues. According to Moal *et al.*, these 16 subjects were matched for age, sex, time (since kidney transplantation) and immunosuppressive treatment (calcineurin inhibitor presence).

The demographic and clinical characteristics of the participating patients (8 chronic hepatitis E virus infection patients and 8 healthy controls) at the time of inclusion are briefly described:

[1] CHE patients, at the time of inclusion, did not receive antiviral treatment;

[2] At inclusion, the control patients were free of acute or chronic viral infection;

[3] Serological determination of human immunodeficiency virus, HBV (hepatitis B virus) surface antigen and HCV (hepatitis C virus) was negative for all patients;

[4] The chronic hepatitis E virus infection patients had a median age of 55 years, ranging from 44 up to 77 years;

[5] CHE patients, since hepatitis E virus infection, had a median time of 44 months, ranging from 12 up to 75 months, while the median time since kidney transplantation was 87 months (ranging from 43 -182 months);

[6] All control patients showed normal blood liver test results

The chronic hepatitis E virus infection patients were sorted into 3 groups: the first category involved patients with mild infection (showed early hepatitis E virus clearance), *i.e.*, within 6 months after inclusion (median time (MT): 4 months; range (R): 1.3-4.6 months), the second category involved patients having moderate infection (showed delayed hepatitis E virus clearance) *i.e.*, >6 months after inclusion (MT: 11.5 months; R: 8.9-17.4 months) and the final category involved patients having severe infection (showed no hepatitis E virus clearance during data analysis time), *i.e.*, >17.4 months after inclusion [15]. The publicly available NCBI GSE series (GSE36539) was already sorted into three groups: mild, moderate & severe. The data of these groups were then preprocessed in R for further analysis using the LIMMA package. Given that the categorization into these 3 groups was already established in the publicly available dataset, no additional statistical analysis was performed to validate this categorization in our study.

## Network topology:

The DEGs were mapped onto the PPI network using >0.7 (as threshold value) interaction score. Subsequently, Cytoscape 3.4 [17] was used to visualize and construct the PPI network. The nodes that possessed greatest numbers of interactions with neighbouring nodes were termed as hub nodes (high degree nodes).

## Network clustering:

The plug-in Molecular Complex Detection (MCODE) was used to identify the densely connected regions of the PPI network in Cytoscape [17]. MCODE was then applied to screen modules of the PPI network with the following parameters: Degree cutoff, 2; node score cutoff, 0.2; k-core, 2; and the maximum depth, 100. Thus, the significant modules were selected from the PPI network.

## Hub gene-associated miRNA elucidation:

After the identification of hub genes (based on centrality approach), miRNet 2.0 [18, 19] platform was utilized to find out the hub gene-associated miRNAs. MiRNet 2.0 assists in elucidating miRNAs using network-based visual analytics.

## Hub miRNA enrichment analysis:

The pathway enrichment analysis of hub miRNAs was implemented using MIENTURNET (MIcroRNA Enrichment TURned NET work) web-tool [20]. MIENTURNET web-tool offers effective prioritization of the miRNA-target interactions, thus assisting researchers (lacking computational skills) avoid navigating multiple websites. As MIENTURNET offer functional enrichment analysis among different annotation categories (*e.g.* KEGG pathways, Reactome Wiki Pathways and Disease Ontology). The selected hub genes-associated miRNAs were further explored for enrichment analysis.

## Results:

## Significant module selection from PPI Network:

According to the previous study, the identified 6 genes were found to be common in all three levels of infection whose expression level increases with the increase in the level of infection (OASL, IFI27, IFIT1, IFIT3, RSAD2 and IFI44L). Though previously identified [14], there were a total of 9 genes common among all three stages, here only 6 of these genes made into the PPI network. It is noteworthy that these six genes form motif with each other. This means that they work together in combination. The PPI network constructed from total DEGs consisted of 129 nodes and 3665 edges (Figure 2 - see PDF). The 3 significant modules, based on degree of importance, were selected from the PPI network and further analyzed using Cytotype MCODE (Figure 3A - (see PDF) and Figure 3B - (see PDF)).

## Module 1:

Seventy nine nodes and 3079 edges constituted Module 1. In the MF group, the genes mainly showed association with the structural constituent of ribosome, RNA binding and rRNA binding. In the BP group, the genes mainly showed association with SRP-dependent co-translational protein targeting to membrane, viral transcription, nuclear-transcribed mRNA catabolic process, nonsense-mediated decay, translational initiation and rRNA processing. In the CC group, the genes mainly showed relation with ribosome, cytosol and focal adhesion. The enrichment analysis of module 1 gene function is mentioned (Table 1).

## Module 2:

Thirty four nodes and 482 edges constituted Module 2. In the MF group, the genes mainly showed association with double-stranded RNA binding, 2'-5'-oligoadenylate synthetase activity, protein binding, transferase activity, nucleotidyl transferase activity. In the BP group, the genes mainly showed association with an interferon signaling pathway, defense response to the virus and negative regulation of viral genome replication. In the CC group, the genes mainly showed relation with cytosol and cytoplasm. The enrichment analysis of module 2 gene function is mentioned (Table 2).

## Module 3:

Sixteen nodes and 104 edges constituted module 3. In the MF group, the genes were mainly related to MHC class II receptor activity, zinc ion binding, ligase activity, protein binding, bridging, peptide antigen binding. In the BP group, the genes were mainly associated with an interferon signaling pathway, positive regulation of sequence-specific DNA binding transcription factor activity, protein trimerization, antigen processing and immune response. In the CC group, the genes were mainly linked to clathrin-coated endocytic vesicle membrane, MHC class II protein complex, Integral component of lumenal side of endoplasmic reticulum membrane and transport vesicle membrane. The enrichment analysis of module 3 gene function is mentioned (Table 3).

## Prediction of hub gene-associated key miRNAs:

The target miRNAs for the obtained six hub genes was predicted in order to identify their role in CHE-associated pathogenesis (Figure 4A - see PDF). The figure (Figure 4B - see PDF) shows the top 15 nodes with 75 edges on the basis of degree extracted from mRNAs-miRNAs network.

## Enrichment analysis of key miRNAs:

The disease ontology of the key miRNAs (miR-129-2-3p, miR-130a-3p, miR-138-5p, miR-212-3p, miR-221-3p, miR-27b-3p and miR-29c-3p) is shown in the upper panel in (Figure 5A - see PDF). Where we submitted 9 key miRNAs to the MIEURTURNET server, which gives the results of 7 miRNAs (excluding two miRNAs). Additionally, KEGG pathways were explored for the key miRNAs from mieunturnet (Figure 5B - see PDF).

## Discussion:

Hepatitis E is a major health issue in both developed and developing nations [21]. In order decrease the risk of chronic infections, it is important to treat the patients (chronically infected) at an early stage which requires timely diagnosis. Patients with kidney transplantation have progressively been reported with chronic hepatitis E virus infection since 2008 [5, 6], but the underlying molecular mechanisms leading to the development of this disease remain obscure/unexplored. Therefore, in this regard, CHE-associated mechanisms require in-depth understanding to identify therapeutic targets of this disease. Molecular components found within human cells are not functionally independent, (*i.e.*, the components are interdependent), which suggests that an individual disease is the outcome of the perturbations of complex intracellular and intercellular interactions. The emerging field of network biology systematically explores key genes, biomarkers or drug targets of the network through modules and pathway identification, thus, serving as platforms for enhanced diagnosis and prognosis as well as treatment of complex diseases [22, 23-24]. Herein, the retrieved NCBI-Gene Expression Omnibus database (NCBI-GEO) human microarray dataset was utilized for the present analysis. The mRNA-miRNA network was explored to provide compelling evidence regarding pathophysiological mechanisms development related to chronic hepatitis E virus infection. The key genes associated miRNAs regulating the network included miR-129-2-3p, miR-130a-3p, miR-138-5p, miR-212-3p, miR-221-3p, miR-27b-3p and miR-29c-3p. Our research might assist in understanding the evolution of chronic hepatitis E virus infection in kidney transplant recipients. Our previous microarray-based report [14] offered significant insight into the pathogenesis of chronic hepatitis E virus infection as identified motif consisting of six genes OASL, IFI27, IFIT1, IFIT3, RSAD2 and IFI44L. This study explored the other important aspect of genes specific to stage transition of hepatitis E virus [14]. The identification of a 6-gene network motif, consisting of OASL, IFI27, IFIT1, IFIT3, RSAD2 and IFI44L, which exhibits increased expression levels with the progression of hepatitis E virus (HEV) infection, sheds light on the intricate molecular mechanisms underlying the host's response to chronic hepatitis E virus infection. Notably, these six genes were found to be common at each stage of infection, forming a motif characterized by their interconnectivity and coordinated function [14].

The formation of the identified motif consisting of the six genes not only reflects the host's concerted effort to combat hepatitis E virus infection but also signifies a unique molecular signature exclusive to this research. Moreover, previous studies have implicated similar ISGs, including OASL, IFI27, IFIT1 and IFI44L, in the pathogenesis of other viral infections such as hepatitis C virus (HCV), suggesting a broader role for these genes in viral-induced liver diseases [15]. In fact, a protein-protein interaction (PPI) network analysis in HCV patients has identified these hub genes as essential components involved in fibrogenesis, further emphasizing their significance in liver disease progression [25, 26, 27, 28- 29]. In the PPI network, module 1 was associated with the ribosome and RNA binding while module 2 was mainly linked to the cytosol and RNA binding. Additionally, module 3 was mainly associated with catalytic activities like ligase, zinc ion binding and other binding activities. The open-reading frame 1 (ORF1) polyprotein in hepatitis E virus constituting multiple domains is majorly attributed to viral replication, which includes methyltransferase domain (Met), Y-domain (Y), papain-like-cysteine protease domain (PCP), hyper variable region domain (HVR), X-domain (X), helicase domain (Hel) and RNA-dependent RNA polymerase domain (RdRp) [28]. Reports have shown association of some of the domain's functional implications to transferase activity, RNA binding, helicase activity, zinc-ion binding and protein binding [29, 30, 31, 32, 34, 35, 36, 37, 38, 39, 40, 41, 42, 43, 44-45]. Therefore, these obtained functions support the hypothesis proposed by the current study. A study has demonstrated that miR-129-2-3p directly targets Wip1 that suppresses the proliferation and invasion of intrahepatic cholangiocarcinoma (ICC). This study has revealed that miR-129-3p plays critical role in ICC pathogenesis and can act as a potential target for its treatment [46]. The role of MiR-129-2-3p as a cancer repressor was also revealed in EC (esophageal carcinoma) cell [47]. Role of miR-130a in both Hepatitis C Virus and Hepatitis B Virus regulation has been revealed [48]. MiR-130a regulates replication through targeting the PKLR gene (gene encoding pyruvate kinase in liver and red blood cell) [48]. The role of miR-138-5p has been revealed in HBV-associated HCC (hepatocellular carcinoma) [49]. It was discovered that miR-138-5p impaired the replication and expression of HBV by down-regulating TNFAIP3 (codes for tumor necrosis factor, alpha-induced protein 3 or A20) [49]. Investigations have demonstrated the crucial role played by miR-138-5p in regulation of various cancers through mediation of biological processes, such as, prostate cancer (combines with FOXC1 [Forkhead box C1]) [50] breast carcinoma (binds to RHBDD1 [rhomboid domain containing 1]) [51], hepatocellular carcinoma (as tumor-suppressing factor) [52]. A study by Shiu and colleagues revealed that miR-138 accelerates HCC cell senescence via repression of TERT (telomerase reverse transcriptase) in HCV-associated HCC [53]. Mir-138-5p represses the development of RB (retinoblastoma) via suppression of PDK1 (pyruvate dehydrogenase kinase 1), that could further assist in explaining the RB tumorigenesis mechanism [54]. MiR-212 functions as a tumor suppressor in NSCLC (non-small cell lung cancer) through SOX4 [55]. While inhibits renal cell cancer proliferation and invasion via suppression of XIAP [56]. The miRNA-212-3p also inhibits the HCC (Human Hepatocellular Carcinoma) proliferation and invasion via suppression of CTGF expression [57]. It was revealed that the overexpression of miR-221-3p was associated with proliferation and HBV amplification [58]. Moreover, the expression of miR-221-3p in HCC tissues was found to significantly elevate [59]. Recent report has demonstrated that HCV infection is mediated by miR-27b through downregulation of LIPC (hepatic lipase C) [60]. It has been documented that miR-27b-3p regulates microglial inflammation response and cell apoptosis through inhibition of A20 (TNF-α-induced protein 3) [61]. Additionally, MiR-29c acts as tumor suppressive miRNA in the growth and advancement of HBV-related HCC by targeting TNFAIP3 (tumor necrosis factor alpha-induced protein 3) [62]. Also, miR-29c plays essential role in HCV infection by targeting STAT3 [63]. The miRNAs including miR-125b-5p, miR-192-5p and miR-99a-5p have been associated with the diagnosis of chronic hepatitis E [64]. Moreover, a study has also revealed the importance of miR-335 in chronic hepatitis [65]. It was reported that hepatitis E virus infection (Genotype 3) is associated with miR-122, miR-885, miR-194, miR-30a, miR-221, miR-223 and miR-27a [65]. Thus, the present discovered key gene-associated-miRNAs including miR-129-2-3p, miR-130a-3p, miR-138-5p, miR-212-3p, miR-221-3p, miR-27b-3p and miR-29c-3p have been reported for the first time. Thus, it could be interpreted that the potentially identified candidate miRNAs and their pathways from this study could offer us an idea of new therapeutic targets for chronic hepatitis E virus infection treatment.

## Conclusions:

We report that miRNAs (miR-129-2-3p, miR-130a-3p, miR-138-5p, miR-212-3p, miR-221-3p, miR-27b-3p and miR-29c-3p) are associated with Hepatitis E virus infection for further validation and analysis.

## Availability of data and material:

Not applicable

## Funding:

Not applicable

## Figures and Tables

**Table 1:** Pathway enrichment analysis of module 1 gene function

**Term**	**Description**	**Genes**	**Count**	**P-value**	**FDR**
**Molecular Function**					
GO:0003735	Structural constituent of ribosome	RPL4, RPL5, RPL30, RPL3, RPL32, RPL31, RPL34, RPLP1, RPL10L, RPLP0, RPL10A, RPL8, RPL9, RPL6, RPL7, RPS4X, RPS15, RPS14, RPL7A, RPS17, RPS16, RPS19, RPL18A, RPS18, RPL36, RPLP2, RPL35, RPL38, RPS11, RPL39, RPS10, RPS13, RPS12, RPS9, RPL21, RPS7, RPS8, RPL23, RPS5, RPL22, RPS6, RPL13A, RPS3A, RPSA, RPL37A, RPL24, RPL27, RPL26, RPL29, RPL28, UBA52, RPL10, RPL12, RPL11, RPS15A, RPS3, RPL14, RPL13, RPS2, RPL15, RPS27A, RPL18, RPL17, RPL19, RPL35A, RPL23A, RPS26, RPS28, RPS27, RPS29, RPL27A, RPS20, FAU, RPS21, RPS24, RPS23	76	4.99E-143	2.55E-141
GO:0044822	Poly(A) RNA binding	RPL4, RPL5, RPL30, RPL3, RPL32, RPL31, RPLP0, RPL10A, RPL8, RPL6, RPL7, RPS4X, RPS15, RPS14, RPL7A, RPS17, RPS16, RPS19, RPL18A, RPS18, RPL36, RPL35, RPS11, RPS10, RPS13, RPS12, RPS9, RPL21, RPS7, RPS8, RPL23, RPS5, RPL22, RPS6, RPL13A, RPS3A, RPSA, RPL37A, RPL24, RPL27, RPL26, RPL29, RPL28, RPL10, RPL12, RPL11, RPS15A, RPS3, RPL14, RPL13, RPS2, RPL15, RPS27A, RPL17, EIF4B, RPL19, RPL35A, RPL23A, RPS26, RPS25, RPS28, RPS27, RPL27A, RPS20, FAU, RPS21, RPS24, RPS23	68	1.16E-67	2.95E-66
GO:0003723	RNA binding	RPL4, RPL5, RPL30, RPL3, RPL31, RPL34, RPL11, RPL10A, RPL8, RPL9, RPL6, RPL7, RPS15, RPS4X, RPS14, RPL7A, RPS15A, RPS16, RPL18A, RPS18, RPL14, RPS3, RPL13, RPL38, RPS2, RPL15, RPL18, RPL39, EIF4B, RPL19, RPL21, RPS7, RPS5, RPL22, RPS3A, RPS25, RPS28, RPL27A, RPL24, RPS20, RPL26, FAU, RPL29, RPL28	44	1.09E-43	1.86E-42
GO:0019843	rRNA binding	RPS4X, RPS9, RPS18, RPS5, RPL12, RPL11, RPL23A, RPL8, RPS11, RPL9	10	1.45E-13	1.85E-12
GO:0070180	Large ribosomal subunit rRNA binding	RPLP1, RPL23, RPLP0, RPLP2, RPL19	5	1.46E-08	1.49E-07
**Biological Process**					
GO:0006614	SRP-dependent cotranslational protein targeting to membrane	RPL4, RPL5, RPL30, RPL3, RPL32, RPL31, RPL34, RPLP1, RPLP0, RPL10A, RPL8, RPL9, RPL6, RPL7, RPS4X, RPS15, RPS14, RPL7A, RPS17, RPS16, RPS19, RPL18A, RPS18, RPL36, RPLP2, RPL35, RPL38, RPS11, RPL39, RPS10, RPS13, RPS12, RPS9, RPL21, RPS7, RPS8, RPL23, RPS5, RPL22, RPS6, RPL13A, RPS3A, RPSA, RPL37A, RPL24, RPL27, RPL26, RPL29, RPL28, UBA52, RPL10, RPL12, RPL11, RPS15A, RPS3, RPL14, RPL13, RPS2, RPL15, RPS27A, RPL18, RPL17, RPL19, RPL35A, RPL23A, RPS26, RPS25, RPS28, RPS27, RPS29, RPL27A, RPS20, FAU, RPS21, RPS24, RPS23	79	1.05E-183	1.99E-181
GO:0019083	Viral transcription	RPL4, RPL5, RPL30, RPL3, RPL32, RPL31, RPL34, RPLP1, RPLP0, RPL10A, RPL8, RPL9, RPL6, RPL7, RPS4X, RPS15, RPS14, RPL7A, RPS17, RPS16, RPS19, RPL18A, RPS18, RPL36, RPLP2, RPL35, PL38, RPS11, RPL39, RPS10, RPS13, RPS12, RPS9, RPL21, RPS7, RPS8, RPL23, RPS5, RPL22, RPS6, RPL13A, RPS3A, RPSA, RPL37A, RPL24, RPL27, RPL26, RPL29, RPL28, UBA52, RPL10, RPL12, RPL11, RPS15A, RPS3, RPL14, RPL13, RPS2, RPL15, RPS27A, RPL18, RPL17, RPL19, RPL35A, RPL23A, RPS26, RPS25, RPS28, RPS27, RPS29, RPL27A, RPS20, FAU, RPS21, RPS24, RPS23	76	1.68E-173	1.59E-171
GO:0000184	Nuclear-transcribed mRNA catabolic process, nonsense-mediated decay	RPL4, RPL5, RPL30, RPL3, RPL32, RPL31, RPL34, RPLP1, RPLP0, RPL10A, RPL8, RPL9, RPL6, RPL7, RPS4X, RPS15, RPS14, RPL7A, RPS17, RPS16, RPS19, RPL18A, RPS18, RPL36, RPLP2, RPL35, RPL38, RPS11, RPL39, RPS10, RPS13, RPS12, RPS9, RPL21, RPS7, RPS8, RPL23, RPS5, RPL22, RPS6, RPL13A, RPS3A, RPSA, RPL37A, RPL24, RPL27, RPL26, RPL29, RPL28, UBA52, RPL10, RPL12, RPL11, RPS15A, RPS3, RPL14, RPL13, RPS2, RPL15, RPS27A, RPL18, RPL17, RPL19, RPL35A, RPL23A, RPS26, RPS25, RPS28, RPS27, RPS29, RPL27A, RPS20, FAU, RPS21, RPS24, RPS23	76	2.45E-170	1.55E-168
GO:0006413	Translational initiation	RPL4, RPL5, RPL30, RPL3, RPL32, RPL31, RPL34, RPLP1, RPLP0, RPL10A, RPL8, RPL9, RPL6, RPL7, RPS4X, RPS15, RPS14, RPL7A, RPS17, RPS16, RPS19, RPL18A, RPS18, RPL36, RPLP2, RPL35, RPL38, RPS11, RPL39, RPS10, RPS13, RPS12, RPS9, RPL21, RPS7, RPS8, RPL23, RPS5, RPL22, RPS6, RPL13A, RPS3A, RPSA, RPL37A, RPL24, RPL27, RPL26, RPL29, RPL28, UBA52, RPL10, RPL12, RPL11, RPS15A, RPS3, RPL14, RPL13, RPS2, RPL15, RPS27A, RPL18, RPL17, EIF4B, RPL19, RPL35A, RPL23A, RPS26, RPS25, RPS28, RPS27, RPS29, RPL27A, RPS20, FAU, RPS21, RPS24, RPS23	77	2.72E-167	1.29E-165
GO:0006364	rRNA processing	RPL4, RPL5, RPL30, RPL3, RPL32, RPL31, RPL34, RPLP1, RPLP0, RPL10A, RPL8, RPL9, RPL6, RPL7, RPS4X, RPS15, RPS14, RPL7A, RPS17, RPS16, RPS19, RPL18A, RPS18, RPL36, RPLP2, RPL35, RPL38, RPS11, RPL39, RPS10, RPS13, RPS12, RPS9, RPL21, RPS7, RPS8, RPL23, RPS5, RPL22, RPS6, RPL13A, RPS3A, RPSA, RPL37A, RPL24, RPL27, RPL26, RPL29, RPL28, UBA52, RPL10, RPL12, RPL11, RPS15A, RPS3, RPL14, RPL13, RPS2, RPL15, RPS27A, RPL18, RPL17, RPL19, RPL35A, RPL23A, RPS26, RPS25, RPS28, RPS27, RPS29, RPL27A, RPS20, FAU, RPS21, RPS24, RPS23	76	2.57E-144	9.78E-143
**Cellular Component**					
GO:0005840	Ribosome	RPL4, RPL5, RPL30, RPL3, RPL32, RPL31, RPL34, RPLP1, RPLP0, RPL10A, RPL8, RPL9, RPL6, RPL7, RPS4X, RPS15, RPS14, RPL7A, RPS17, RPS16, RPS19, RPL18A, RPS18, RPL36, RPLP2, RPL35, RPL38, RPS11, RPS10, RPS13, RPS9, RPL21, RPS7, RPS8, RPL23, RPL22, RPS6, RPL13A, RPS3A, RPL37A, RPL24, RPL27, RPL29, RPL28, UBA52, RPL10, RPL11, RPS15A, RPS3, RPL14, RPL13, RPS2, RPL15, RPS27A, RPL18, RPL19, RPL35A, RPL23A, RPS26, RPS25, RPS28, RPS27, RPS29, RPL27A, RPS20, FAU, RPS21, RPS24, RPS23	69	2.44E-134	8.29E-133
GO:0022625	Cytosolic large ribosomal subunit	RPL4, RPL5, RPL30, RPL3, RPL32, RPL10, RPL31, RPL34, RPLP1, RPL12, RPL10L, RPLP0, RPL11, RPL10A, RPL8, RPL9, RPL6, RPL7, RPL7A, RPL18A, RPL36, RPL14, RPL35, RPLP2, RPL13, RPL38, RPL15, RPL18, RPL39, RPL17, RPL19, RPL21, RPL23, RPL22, RPL35A, RPL13A, RPL23A, RPL27A, RPL37A, RPL24, RPL27, RPL26, RPL29, RPL28, UBA52	45	1.98E-93	3.36E-92
GO:0022627	Cytosolic small ribosomal subunit	RPS15, RPS4X, RPS14, RPS17, RPS15A, RPS16, RPS19, RPS18, RPS3, RPS2, RPS27A, RPS11, RPS10, RPS13, RPS12, RPS9, RPS7, RPS8, RPS5, RPS6, RPSA, RPS3A, RPS26, RPS25, RPS28, RPS27, RPS29, RPS20, FAU, RPS21, RPS24, RPS23	32	1.52E-64	1.72E-63
GO:0005829	Cytosol	RPL4, RPL5, DOCK4, RPL30, RPL3, RPL32, RPL31, RPL34, RPLP1, RPLP0, RPL10A, RPL8, RPL9, RPL6, RPL7, RPS4X, RPS15, RPS14, RPL7A, RPS17, RPS16, RPS19, RPL18A, RPS18, RPL36, RPLP2, RPL35, RPL38, RPS11, RPL39, RPS10, RPS13, RPS12, RPS9, RPL21, RPS7, RPS8, RPL23, RPS5, RPL22, RPS6, RPL13A, RPS3A, RPSA, RPL37A, RPL24, RPL27, RPL26, RPL29, RPL28, UBA52, RPL10, RPL12, RPL11, RPS15A, RPS3, RPL14, RPL13, RPS2, RPL15, RPS27A, RPL18, RPL17, EIF4B, RPL19, RPL35A, RPL23A, RPS26, RPS25, RPS28, RPS27, RPS29, RPL27A, RPS20, FAU, RPS21, RPS24, RPS23	78	3.15E-56	2.68E-55
GO:0005925	Focal adhesion	RPL4, RPL5, RPL30, RPL3, RPL31, RPLP1, RPL12, RPLP0, RPL10A, RPL8, RPL9, RPL6, RPL7, RPS15, RPS4X, RPS14, RPL7A, RPS17, RPS16, RPS19, RPS18, RPS3, RPLP2, RPL38, RPS2, RPS11, RPL18, RPS10, RPS13, RPL19, RPS9, RPS7, RPS8, RPL23, RPS5, RPL22, RPL13A, RPS3A, RPS29, RPL37A, RPL27	41	3.05E-46	2.08E-45
**KEGG_PATHWAY**					
hsa03010	Ribosome	RPL4, RPL5, RPL30, RPL3, RPL32, RPL31, RPL34, RPLP1, RPL10L, RPLP0, RPL10A, RPL8, RPL9, RPL6, RPL7, RPS4X, RPS15, RPS14, RPL7A, RPS17, RPS16, RPS19, RPL18A, RPS18, RPL36, RPLP2, RPL35, RPL38, RPS11, RPL39, RPS10, RPS13, RPS12, RPS9, RPL21, RPS7, RPS8, RPL23, RPS5, RPL22, RPS6, RPL13A, RPS3A, RPSA, RPL37A, RPL24, RPL27, RPL26, RPL29, RPL28, UBA52, RPL10, RPL12, RPL11, RPS15A, RPS3, RPL14, RPL13, RPS2, RPL15, RPS27A, RPL18, RPL17, RPL19, RPL35A, RPL23A, RPS26, RPS25, RPS28, RPS27, RPS29, RPL27A, RPS20, FAU, RPS21, RPS24, RPS23	77	4.38E-138	3.51E-137

**Table 2:** Pathway enrichment analysis of module 2 gene function

**Term**	**Description**	**Genes**	**Count**	**P-value**	**FDR**
**Molecular Function**					
GO:0003725	double-stranded RNA binding	IFIH1, OAS1, DDX58, OAS2, OAS3, DDX60, OASL	7	1.19E-09	5.34E-08
GO:0001730	2'-5'-oligoadenylate synthetase activity	OAS1, OAS2, OAS3, OASL	4	2.24E-08	5.04E-07
GO:0005515	Protein binding	IFITM3, RTP4, IFITM1, IFI6, ADAR, IFI35, IFIT1, DDX60, USP18, IFIT3, IFIT2, IFIH1, HERC5, GBP2, RSAD2, DDX58, STAT1, STAT2, MX2, MX1, ISG15, BST2, OAS1, OAS2, OAS3, IRF7, IRF9	27	2.22E-04	0.003332
GO:0016740	Transferase activity	OAS1, OAS2, OAS3, OASL	4	7.14E-04	0.008029
GO:0016779	Nucleotidyltransferase activity	OAS1, OAS2, OAS3	3	0.001113	0.010019
**Biological Process**					
GO:0060337	Type I interferon signaling pathway	IFITM3, IFITM1, IFITM2, IFI6, ADAR, IFI35, IFIT1, IFIT3, IFIT2, OASL, GBP2, RSAD2, STAT1, STAT2, MX2, MX1, ISG15, BST2, OAS1, IFI27, OAS2, OAS3, IRF7, XAF1, IRF9	25	2.36E-53	3.87E-51
GO:0051607	Defense response to virus	IFITM3, IFITM1, IFITM2, RSAD2, STAT1, STAT2, MX2, MX1, ISG15, ADAR, IFIT1, DDX60, IFIT3, IFI44L, IFIT2, OASL, BST2, HERC5, OAS1, OAS2, OAS3, IRF9	22	5.93E-35	4.87E-33
GO:0009615	Response to virus	IFITM3, IFITM1, IFITM2, RSAD2, DDX58, MX2, MX1, IFI44, ADAR, IFIT1, DDX60, IFIT3, IFIT2, OASL, IFIH1, BST2, OAS1, OAS2, OAS3, IRF7	20	4.76E-34	2.60E-32
GO:0045071	Negative regulation of viral genome replication	IFITM3, BST2, IFITM1, IFITM2, RSAD2, OAS1, OAS3, MX1, ISG15, ADAR, IFIT1, OASL	12	5.76E-22	2.36E-20
GO:0035456	Response to interferon-beta	IFITM3, BST2, IFITM1, IFITM2, STAT1, XAF1	6	2.67E-12	8.77E-11
**Cellular Component**					
GO:0005829	Cytosol	DDX58, STAT1, STAT2, MX2, MX1, ISG15, IFI35, IFIT1, USP18, IFIT3, IFIT2, OASL, IFIH1, HERC5, OAS1, OAS2, OAS3, IRF7, GBP2, XAF1, IRF9, HERC6	22	9.96E-09	4.28E-07
GO:0005737	Cytoplasm	RTP4, DDX58, STAT1, STAT2, MX2, MX1, IFI44, ADAR, IFIT1, DDX60, IFIT3, IFI44L, IFIT2, OASL, BST2, HERC5, OAS1, OAS2, OAS3, IRF7, IRF9, HERC6	22	3.07E-05	6.61E-04
GO:0048471	Perinuclear region of cytoplasm	HERC5, STAT1, OAS2, MX1, GBP2	5	0.024978	0.325655
GO:0005739~	Mitochondrion	IFI27, RSAD2, OAS1, OAS2, IFI6, XAF1, IFIT3	7	0.030293	0.325655
GO:0005634	Nucleus	STAT1, STAT2, MX2, MX1, ADAR, IFI35, USP18, IFIH1, HERC5, OAS1, OAS2, IRF7, XAF1, IRF9, HERC6	15	0.082361	0.708305
**KEGG_PATHWAY**					
hsa05164	Influenza A	IFIH1, RSAD2, OAS1, DDX58, STAT1, OAS2, STAT2, OAS3, MX1, IRF7, ADAR, IRF9	12	1.47E-16	2.35E-15
hsa05162	Measles	IFIH1, OAS1, DDX58, STAT1, OAS2, STAT2, OAS3, MX1, IRF7, ADAR, IRF9	11	1.41E-15	1.13E-14
hsa05168	Herpes simplex infection	IFIH1, OAS1, DDX58, STAT1, OAS2, STAT2, OAS3, IRF7, IFIT1, IRF9	10	3.58E-12	1.91E-11
hsa05160	Hepatitis C	OAS1, DDX58, STAT1, OAS2, STAT2, OAS3, IRF7, IFIT1, IRF9	9	1.88E-11	7.51E-11
hsa05161	Hepatitis B	IFIH1, DDX58, STAT1, STAT2, IRF7	5	1.17E-04	3.74E-04

**Table 3:** Pathway enrichment analysis of module 3 gene function

**Term**	**Description**	**Genes**	**Count**	**P-value**	**FDR**
**Molecular Function**					
GO:0032395	MHC class II receptor activity	HLA-DQA2, HLA-DQB2, HLA-DQA1, HLA-DQB1	4	2.57E-07	8.47E-06
GO:0008270	Zinc ion binding	SP100, MT2A, TRIM6, TRIM5, TRIM14, TRIM38, TRIM21, TRIM22, PML	9	2.14E-06	3.53E-05
GO:0016874	Ligase activity	TRIM6, TRIM5, TRIM38, TRIM21, TRIM22	5	7.60E-05	8.36E-04
GO:0030674	Protein binding, bridging	TRIM6, TRIM5, TRIM22	3	0.002075	0.017118
GO:0042605	Peptide antigen binding	HLA-DQA1, HLA-DQB1	2	0.024603	0.146059
**Biological Function**					
GO:0060333	Interferon-gamma-mediated signaling pathway	SP100, MT2A, TRIM5, TRIM38, TRIM21, FCGR1B, HLA-DQA2, HLA-DQB2, TRIM22, PML, HLA-DQA1, HLA-DQB1	12	4.61E-24	6.45E-22
GO:0051091	Positive regulation of sequence-specific DNA binding transcription factor activity	SP100, TRIM5, TRIM14, TRIM38, TRIM21, TRIM22	6	2.48E-08	1.74E-06
GO:0070206	Protein trimerization	TRIM6, TRIM5, TRIM21, TRIM22	4	4.83E-08	2.25E-06
GO:0002504	Antigen processing and presentation of peptide or polysaccharide antigen via MHC class II	HLA-DQA2, HLA-DQB2, HLA-DQA1, HLA-DQB1	4	3.89E-07	1.36E-05
GO:0006955	Immune response	FCGR1B, HLA-DQA2, HLA-DQB2, TRIM22, HLA-DQA1, HLA-DQB1	6	2.36E-05	6.61E-04
**Cellular Component**					
GO:0030669	Clathrin-coated endocytic vesicle membrane	FCGR1B, HLA-DQA2, HLA-DQB2, HLA-DQA1, HLA-DQB1	5	2.96E-08	8.57E-07
GO:0042613	MHC class II protein complex	HLA-DQA2, HLA-DQB2, HLA-DQA1, HLA-DQB1	4	6.88E-07	9.98E-06
GO:0071556	Integral component of lumenal side of endoplasmic reticulum membrane	HLA-DQA2, HLA-DQB2, HLA-DQA1, HLA-DQB1	4	1.63E-06	1.57E-05
GO:0030658	Transport vesicle membrane	HLA-DQA2, HLA-DQB2, HLA-DQA1, HLA-DQB1	4	3.74E-06	2.71E-05
GO:0012507	ER to Golgi transport vesicle membrane	HLA-DQA2, HLA-DQB2, HLA-DQA1, HLA-DQB1	4	9.73E-06	5.64E-05
**KEGG_PATHWAY**					
hsa05168	Herpes simplex infection	SP100, HLA-DQA2, PML, HLA-DQA1, HLA-DQB1	5	6.98E-06	1.95E-04
hsa05322	Systemic lupus erythematosus	TRIM21, HLA-DQA2, HLA-DQA1, HLA-DQB1	4	1.38E-04	0.001852
hsa05310	Asthma	HLA-DQA2, HLA-DQA1, HLA-DQB1	3	2.73E-04	0.001852
hsa05164	Influenza A	HLA-DQA2, PML, HLA-DQA1, HLA-DQB1	4	3.01E-04	0.001852
hsa05332	Graft-versus-host disease	HLA-DQA2, HLA-DQA1, HLA-DQB1	3	3.31E-04	0.001852
